# Public involvement in big data projects: an ethnographically-informed study.

**DOI:** 10.23889/ijpds.v7i3.1991

**Published:** 2022-08-25

**Authors:** Elisa Jones, Lucy Frith, Anna Chiumento, Sarah Rodgers, Alan Clarke, Sarah Markham

**Affiliations:** 1 University of Liverpool; 2 University of Manchester; 3 Public Advisor for the project

## Objectives

Public involvement and engagement (PIE)) is playing an increasingly important role in big data initiatives and projects. It is therefore important to gain a deeper understanding of the different approaches used.

## Approach

This study explores PIE using ethnographically-informed qualitative case studies. The case studies include: three citizen juries, each one carried out over eight days and that asked jurors to consider different real-world health data initiatives; and a public panel set up by a regional databank that carries out data linking. Data collection is ongoing and I will be continuing to carry out close observations of activities, and conducting semi-structured 1:1 interviews with those that organise and have taken part in the activities.

## Results

Data collection so far comprises completed observations at the citizen juries (~96 hours), ongoing observations of the public panel meetings (~15 hours), and thirty semi-structured 1:1 interviews with public contributors and other stakeholders about their experiences of the activities they were involved in. Early data analysis indicates key themes of: jurors feeling heard, but unsure whether anybody was listening; stakeholders being impressed by informed jurors, but raising concerns over contributors becoming too ‘expert’; how who is at the table and what information is presented impacts what is discussed; differences between online and in-person participation; and public involvement not being a substitute for informing the public about how their data is used.

## Conclusion

‘Who’ is involved, and ‘how’ PPIE activities are designed and run can facilitate or constrain discussion, enhancing or limiting public contributions. If public involvement is to achieve its aims, including increasing trustworthiness, deeper consideration of these factors by those who seek the public’s views in their data projects is recommended.

